# Elucidation of the anti-colorectal cancer mechanism of *Atractylodes lancea* by network pharmacology and experimental verification

**DOI:** 10.18632/aging.206075

**Published:** 2024-08-22

**Authors:** Guangliang Wang, Chuangchuang Guo, Hui Pi, Yu Wang, Shuyun Lin, Keyi Bi, Ming Zhang, Na Wang, Guojun Zhao

**Affiliations:** 1 Affiliated Qingyuan Hospital (Qingyuan People’s Hospital), Guangzhou Medical University, Qingyuan 511518, Guangdong, China; 2 Department of Histology and Embryology, Faculty of Basic Medical Sciences, Guilin Medical University, Guilin 541000, Guangxi, China; 3 Faculty of Public Health, Guilin Medical University, Guilin 541000, Guangxi, China; 4 Faculty of Basic Medical Sciences, Dali University, Dali 671003, Yunnan, China; 5 Department of Pharmacy, Guilin Medical University, Guilin 541000, Guangxi, China

**Keywords:** colorectal cancer, *Atractylodes lancea*, network pharmacology, molecular docking, luteolin

## Abstract

*Atractylodes lancea* which was listed in “Shennong’s Materia Medica” and could be used to treat gastrointestinal-associated diseases. However, its roles, core and active ingredients, and mechanisms in treatment of colorectal cancer (CRC) were still unknown. Therefore, network pharmacology and experimental validation were used to clarify the effects, core active ingredients and molecular mechanisms of *Atractylodes lancea*. We found that *Atractylodes lancea* has 28 effective active components and 213 potential targets. Seventy-three genes which demonstrate interaction between the *Atractylodes lancea* and CRC were confirmed. Enrichment analysis showed that 2033 GO biological process items and 144 KEGG pathways. Survival and molecular docking analysis revealed that luteolin as the core component interacted with these genes (Matrix metalloproteinase 3 (MMP3), Matrix metalloproteinase 9 (MMP9), Tissue inhibitor of metalloproteinases 1 (TIMP1), Vascular endothelial growth factor A (VEGFA)) with the lowest binding energy, and these genes were involved in building a prognostic model for CRC. Cellular phenotyping experiments showed that luteolin could inhibit the proliferation and migration of CRC cells and downregulate the expression of MMP3, MMP9, TIMP1, VEGFA probably by Phosphoinositide 3-kinase/ serine/threonine kinase Akt (PI3K/AKT) pathway. To conclude, *Atractylodes lancea* could inhibit proliferation and migration of CRC cells through its core active ingredient (luteolin) to suppress the expression of MMP3, MMP9, TIMP1, VEGFA probably by PI3K/AKT pathway.

## INTRODUCTION

Colorectal cancer (CRC) is one of the most commonly diagnosed digestive cancers in the world. About 60-70% of CRC patients were diagnosed at the late stage [1]. 25%-30% of patients present with metastases [2]. A series of factors that lead to the development of CRC include obesity, smoking and eating red meat [3]. At present, the methods treatment for CRC is still surgery, supplemented by chemotherapy and radiotherapy [4]. Currently, traditional chemotherapeutic drugs inhibit and kill no matter of tumor cells, but normal cells, moreover, due to the effects of toxic side and drugs resistance of tumor, many drugs are limited to clinical applications [4]. Therefore, it was necessary to discover new drugs to achieve the high efficiency anti-tumor ability with low toxicity. In traditional Chinese medicine, some plants with the characteristics of low-toxicity anti-tumor properties. This study was aimed to provide theoretical support to develop these low toxicity, plant-based drugs.

Phytochemicals, as natural substances in plants, are important resources for the development of novel anti-tumor drugs. Research and clinical studies assume that phytochemicals have anti-carcinogenic effects, including inhibiting proliferation, inducing apoptosis, and enhancing the excretion of carcinogens [5]. *Atractylodes lancea* (Chinese: Cangzhu) was a widely used traditional Chinese medicine (TCM) and listed in “Shennong’s Materia Medica”, the rhizome tastes pungent and bitter, and was attributed to the spleen, stomach and liver meridians. According to the theory of TCM, *Atractylodes lancea* could be used to treat rheumatic diseases, digestive disorders, night blindness, and influenza. *Atractylodes lancea* was generally used in Ermiao Powder, Simiao Powder and so on in traditional decoctions. Recently, it was found that *Atractylodes lancea* extracts also have anti-cancer and anti-inflammatory effects [6]. Additionally, recent studies have shown that *Atractylodes lancea* could inhibit proliferation by cellular signaling pathways involved in gastric cancer cells [7], Cholangiocarcinoma (CCA) [8], non-small cell lung cancer [9] and so on. Among these researches, it has been reported the ethanolic extract of *Atractylodes lancea* inhibits the progress of Opisthorchis viverrini/dimethylnitrosamine (DMN)-induced CCA model and CCA-xenografted nude mice without significant toxicity, compared with 5-FU and the untreated control [10, 11]; phase I and II clinical trial have shown that it can improve clinical response and disease progression of CCA patients [12, 13]. In CRC studies, as adjunctive therapy, *Atractylodes lancea* could relieve nausea, vomiting and neutropenia after Oxaliplatin treatment [14, 15]; mixtures consisting of *Atractylodes lancea* were significantly inhibited metastasis of CRC in murine lung-metastasis model [16]; 13 randomized controlled trials were analyzed and found that *Atractylodes lancea* could have contributed to improve CRC response [17]. However, the role, core active ingredients and mechanism of *Atractylodes lancea* in the treatment of CRC are currently less studied.

In drug development, developing multi-targeted drugs for complex diseases such as cancer was rapidly growing. Network pharmacology could systematically observe the impact of drugs on diseases, thereby revealing the complexity of Chinese medicine and disease through the interaction of “disease-gene-protein-drug” [18]. Studies have shown that network pharmacology has been used to reveal the potential activity of ingredients, targets and mechanisms of Chinese medicine in the treatment of disease, and to provide new strategies to develop new drugs [19]. In this study, we found that luteolin as the core active component of *Atractylodes lancea* plays an anti-CRC role and its molecular mechanism against CRC by network pharmacology and experimental verification.

## MATERIALS AND METHODS

### Target collection for *Atractylodes lancea*

Pharmacological platforms such as HIT [20], TCMSP [21] and TCMID [22] were utilized to search for the active components of *Atractylodes lancea*. The results of the search were combined to remove the compounds that were duplicate and did not have PubChem ID. According to the PubChem ID, the target gene corresponding to the active ingredient was retrieved in HIT.

### Identify critical genes for CRC from disease databases

Using “colorectal cancer” and “colorectal adenoma” as search terms, we searched the GeneCards [23], OMIM [24] and DigSee [25] databases for potential targets which related to CRC. Targets from these three disease databases were combined and duplicate values were removed to obtain CRC related targets.

### Identify key genes in CRC from expression data

We obtained expression data separately from Gene Expression Omnibus (GEO) and The Cancer Genome Atlas (TCGA) databases. GSE164191 was obtained from the GEO database (http://www.ncbi.nlm.nih.gov/geo/) and includes expression profiles of 59 CRC peripheral blood samples and 62 normal peripheral blood samples. Limma package [26] was used to check raw gene expression data. Volcano plots of CRC VS Normal were created to indicate significantly up- and down-regulated genes. Genes with adjusted p-value less than 0.05 were screened and considered as Differential Expression Genes (DEGs). The top 100 DEGs obtained from the above process were used for further studies. Heatmap of the dataset was plotted using SRplot (https://www.bioinformatics.com.cn/) and network of DEGs was plotted using Cytoscape.

### PPI network construction

Targets obtained from disease database were integrated and Venn diagram [27] was plotted to identify genes shared by targets of *Atractylodes lancea* and CRC. Protein-protein interaction (PPI) networks were created using STRING [28] (http://string-db.org). Interaction scores were set at 0.4 ~ 10. The hub genes and crucial modules of the *Atractylodes lancea* anti-CRC from PPI network were calculated using the MCC algorithm in the CytoHubba plug-in for CytoScape, and then the network of related protein targets was constructed. Finally, the top 10 core targets were also displayed.

### Pathway and functional enrichment analysis

In order to clarify the roles of target proteins interacting with the target genes of *Atractylodes lancea* in gene functions and signaling pathways, we performed Gene Ontology (GO) and Kyoto Encyclopedia of Genes and Genomes (KEGG) enrichment analysis of potential targets of *Atractylodes macrocephala* for intervention in CRC using clusterProfiler [29]. The enriched GO terms and pathways with *p*-values less than 0.05 were selected for further visualization.

### Correlation, expression and survival analysis

The correlation and expression among the hub genes were analyzed using Gene Expression Profiling Interactive Analysis (GEPIA). By multifactorial Cox regression analysis, the prognostic model constructed by the hub genes was able to accurately and efficiently classify CRC into different risk categories, and the survival of low-risk CRC patients was significantly longer than that of high-risk CRC patients. Meanwhile, the Area Under Curve (AUC) values for the prognostic model predicting 1-, 3-, and 5-year survival were determined by Receiver Operator Curves (ROC) curve analysis.

### Molecular docking

The target genes corresponding to each active ingredient in the Chinese medicines were intersected with the intersection genes respectively, and the one with the most intersections was taken as the core ingredient of the drug. Molecular docking was performed in order to predict the interactions between these genes and *Atractylodes lancea* [30]. The structures of the target proteins were downloaded from the Protein Data Bank (PDB) database (https://www.rcsb.org). Water molecules and original ligands were removed from the protein structures using PyMOL. Proteins were further processed and docked using AutoDock Tools 1.5.6. Visualizations of the 3D interactions between the top four protein-*Atractylodes lancea* complexes with the lowest binding energies were built using PyMOL [31].

### Cell culture

CRC cell lines NCM460, RKO, HCT15, SW480, SW620 and HT29 were purchased from Cell Bank of the Chinese Academy of Sciences (Shanghai, China), and were cultured in DMEM medium and RPMI1640 medium, respectively. All media supplement with 10% fetal bovine serum.

### Cell viability assay

Collected log-phase cells, planted 2000 cells/well into 96-well and cultured. After incubation at 37° C for 24 h, the cells were treated with different concentrations of *Atractylodes lancea* at different times. Next, add 10 μL of Cell Counting Kit (CCK)-8 reagent to each well and continue incubation for 4 h. Then, the optical density (OD) values were measured at 450 nm with an enzyme meter.

### EdU assay

Cells were cultured on coverslips and treated with 40 μM *Atractylodes lancea* for 24 h. Then cells were incubated with 5-ethynyl-2’-deoxyuridine (EdU) working solution for 2 h. Washed the cells with PBS, and incubated with stationary liquid for 30 min at room temperature, washed and permeabilizated (PBS containing 0.3% TritonX-100) for 10 min. After washing with PBS, click reaction solution was added and reacted for 1-2 hours away from light. Next, washed with PBS and added Hoechst 33,342 Reagent for 10 min. EdU-positive cells were measured under confocal microscopy.

### Colony formation experiment

2000 cells/well were planted into 6-well with indicated treatment. Changed the medium and added drugs every two days. After 2 weeks, the colonies were clearly visible. Then the colonies were fixed with 4% paraformaldehyde, stained with crystal violet and photographed.

### Wound-healing assay

When the density of cultured cells reached 90% confluence, a sterile pipette tip was used to produce wound. Cell culture medium was replaced with serum-free DMEM, and migration distance was observed and calculated using a microscope after 72 h.

### RNA isolation and real-time PCR

TRlzol reagent was used to lysis to obtain total RNA according to manufacturer’s instructions. Relevant mRNA expression levels were measured by qRT-PCR assay using SYBR Green PCR Master Mix (Takara, Japan, RR820A). The mRNA levels were normalized to 18s mRNA. Primer sequences were shown in Supplementary Table 1.

### Western blot analysis

Lysis buffer was used to lyse cells, equal amounts of proteins were uploaded and electro-transferred to polyvinylidene fluoride (PVDF) membranes. 5% skim milk was used to block membranes for 2 h at room temperature and then primary antibodies (anti-MMP3 (AF0217, Affinity, USA, 1:2000), anti-MMP9 (AF5228, Affinity, 1:2000), anti-PI3K (A4992, Abclonal, USA, 1:500), anti-p-PI3K (17366, CST, USA, 1:1000), anti-AKT (9272, CST, 1:1000), anti-p-AKT (4060, CST, 1:1000) or anti-GAPDH (5174, CST, 1:1000)) incubated membranes at 4° C overnight. Membranes were incubated with HRP-conjugated secondary antibody (1:1000, SeraCare, USA) for 2 h at room temperature and visualized using the Bio-Rad Image Detection System.

### Statistical analysis

Data were analyzed using SPSS version 18.0. Data are presented as means ± standard deviation (SD) of at least three independent experiments. Normally distributed data sets were analyzed using an unpaired Student’s t-test for two independent groups or a paired t-test for two dependent groups. *p-*values of < 0.05 were considered statistically significant, and *p-*values < 0.05, 0.01, and 0.001, were indicated by one, two, and three asterisks, respectively.

### Data availability statement

The data supporting this study are available from the corresponding authors upon reasonable request.

## RESULTS

### Acquisition of the targets of *Atractylodes lancea*

The HIT, TCMSP and TCMID database were used to search for the core active ingredients of the *Atractylodes lancea*, 72 active ingredients were collected (Supplementary Table 2). According to the PubChem ID, the target genes which corresponding to active ingredients were searched in HIT, the active ingredient without target genes in the database was removed, and finally we got 213 target genes corresponding to 28 active ingredients (Supplementary Table 3).

### Target screening for CRC

**Figure 1:**
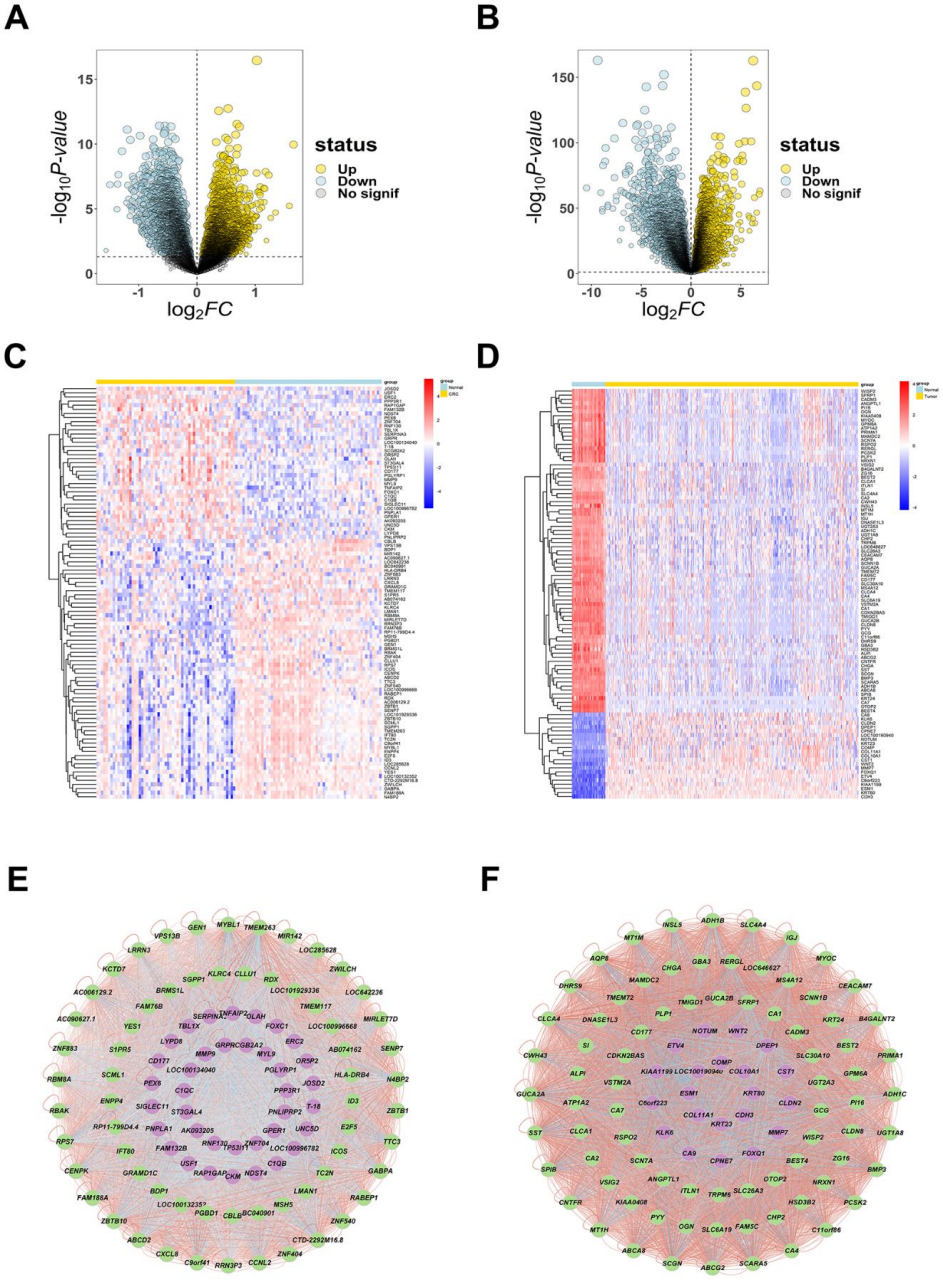
**The DEGs of CRC.** (**A**, **B**) The volcano plots were created to visualize differentially up- and down- regulated genes from GSE164191 and TCGA with significance p < 0.05, reseparate, up-regulated genes are shown in yellow color, down-regulated genes are shown in light blue color. (**C**, **D**) The heatmaps were to show the top 100 up- and down-expressed genes, up-regulated genes are shown in red color, down-regulated genes are shown in blue color. (**E**, **F**) Network diagram (arranged according to LogFC value) of top 100 DEGs were plotted to evaluate their correlation ship, up-regulated genes are shown in purple color, down-regulated genes are shown in green color, significant negative correlation ship are shown in blue lines, significant positive correlation ship are shown in red lines.

By searching the following databases: Genecards, OMIM, DigSee, 11535 genes related with CRC were collected (Supplementary Table 4). Considering the diagnostic and prognostic risk assessment, the peripheral blood datasets GSE164191 from CRC patients was utilized for analysis. 5239 upregulated and 3668 downregulated DEGs were obtained by applying the cut-off adj p-value < 0.05 to the datasets, respectively (Supplementary Table 5). And 6833 up-regulated and 8029 down-regulated genes were obtained in the same way (Supplementary Table 6). These DEGs in both data were used for volcano plots (Figure 1A, 1B), and the top 100 of them were displayed in heatmap (Figure 1C, 1D). The correlation between top 100 DEGs was visualized by using Cytoscape (Figures 1E, 1F).

### Venn diagram and PPI network construction

**Figure 2:**
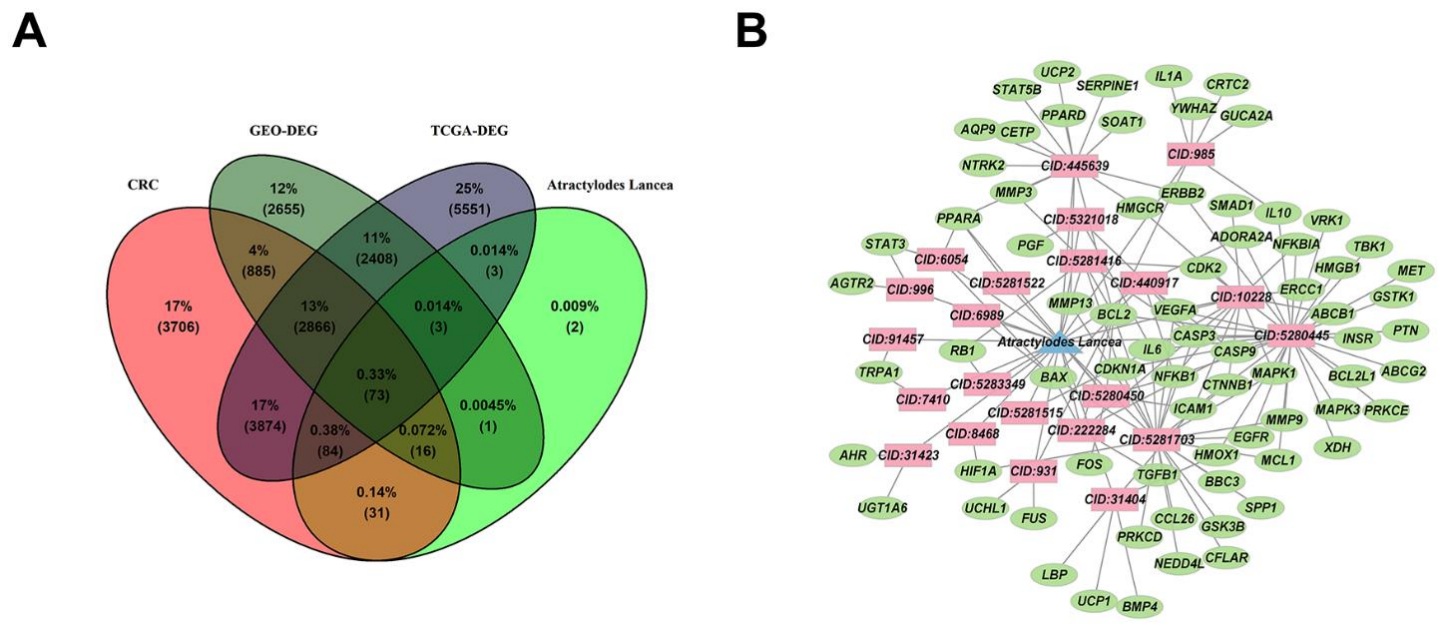
**The targets and relationship between *Atractylodes lancea* and CRC.** (**A**) The co-expression of 73 genes between targets of *Atractylodes lancea* and CRC were overlapped in Venn map. (**B**) Cytoscape constructs a network of relationships between 73 genes and active ingredients of *Atractylodes lancea*, active ingredients are shown in red color, *Atractylodes lancea* are shown in blue color, genes are shown in green color.

The intersection of *Atractylodes lancea*-related target genes and CRC-related target genes was taken, and Venn map was drawn by VennDiagram (Version 1.7.3) to obtain 73 intersection targets (Figure 2A and Supplementary Table 7). Construction of relationship network for 73 genes and *Atractylodes lancea* active ingredients was done by using Cytoscape (Figure 2B).

**Figure 3:**
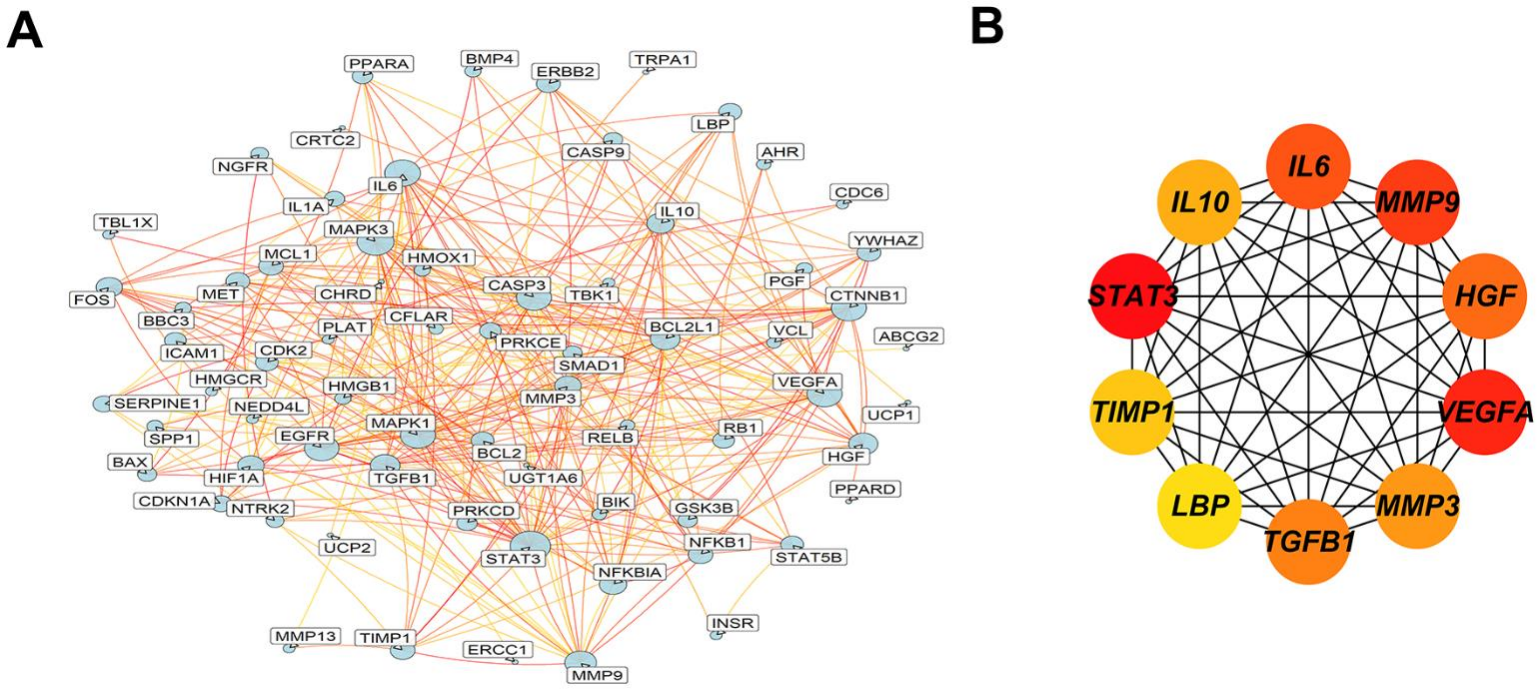
**Protein–protein interaction network diagram of 73 intersected DEGs.** (**A**) An interaction network of 73 overlap genes was constructed by Cytoscape, larger point means larger degree. (**B**) The top 10 hub genes were identified and displayed a network by MCC algorithm, in which gray value represents the importance in the network.

The overlapped targets were submitted to STRING for PPI network construction, and the interacting proteins with a score cutoff > 0.4 and size cutoff < 10 were selected. Finally, 304 interaction relationships were collected and visualized by Cytoscape (Figure 3A). By using Cytohubba, the plug-in of Cytoscape, the hub genes were displayed in the interaction network. The top 10 hub genes of target proteins were identified, and a network was constructed. We found that STAT3, VEGFA, MMP9, IL6, HGF, TGFB1, MMP3, IL10, TIMP1, LBP were the important targets for *Atractylodes lancea* to intervene CRC (Figure 3B).

### GO and KEGG analysis

**Figure 4:**
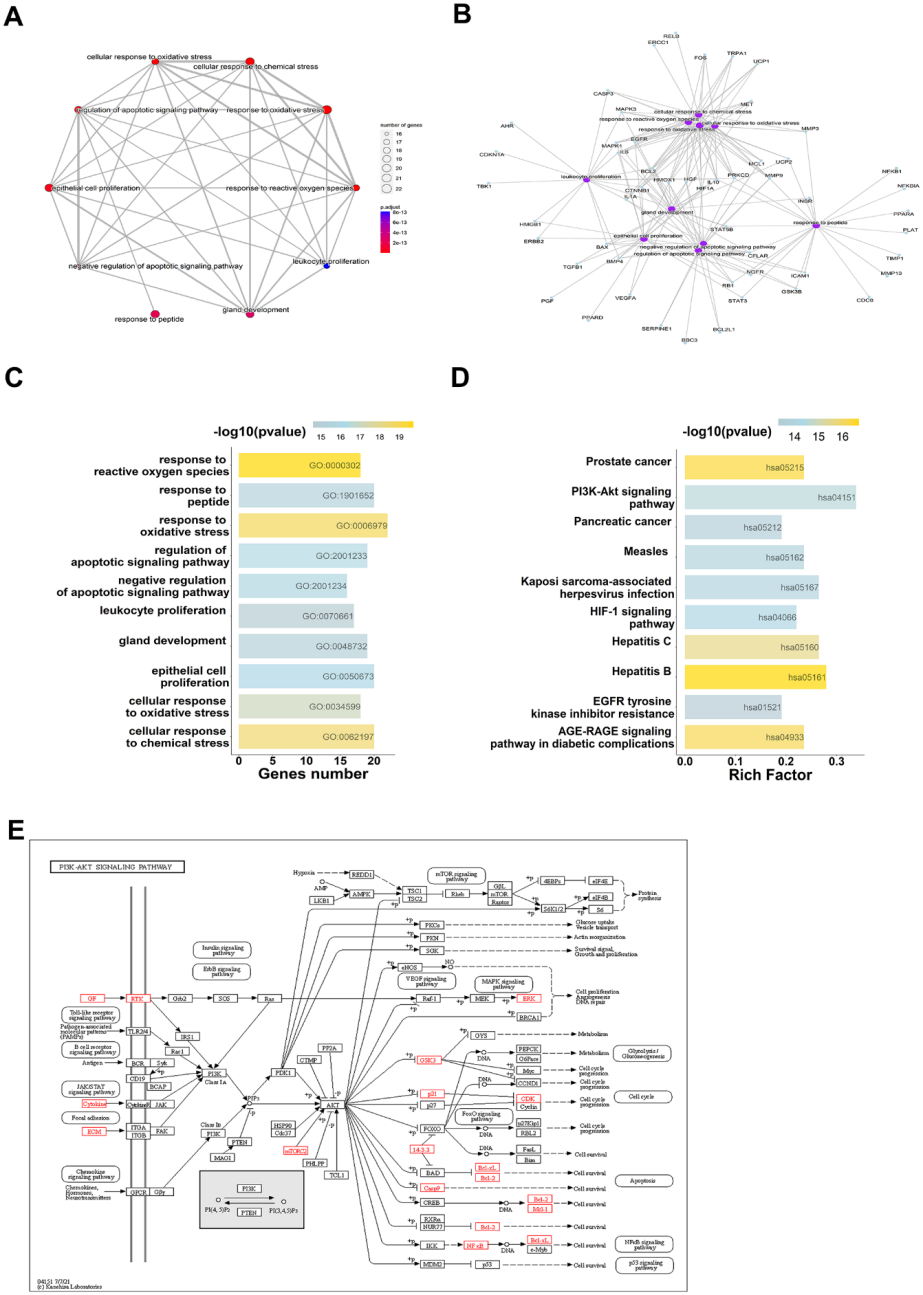
**GO and KEGG analysis show the 73 overlap genes targeted by *Atractylodes lancea*.** (**A**–**C**) GO enrichment analysis shows the intersection target genes in BP and the top 10 results were displayed. (**D**, **E**) KEGG pathway analysis shows the intersection target genes, top 10 results and the richest factor enrichment pathway - PI3K/AKT signaling pathway were displayed.

To explore the possible mechanisms of the anti-CRC effect of *Atractylodes lancea*, 73 intersection targets were used to analysis GO and KEGG enrichment with R software and the results were visualized. GO enrichment analysis showed that a total of 2033 biological processes (BP) items, and the top 10 significantly enriched terms were displayed, according to p < 0.05 (Supplementary Table 8). The target proteins were involved in response to reactive oxygen species, response to oxidative stress, cellular response to chemical stress, cellular response to oxidative stress, regulation of apoptotic signaling pathway, epithelial cell proliferation, negative regulation of apoptotic signaling pathway, response to peptide, gland development, leukocyte proliferation (Figure 4A–4C). The top 10 significantly enriched terms of cellular components localization were primarily associated with extracellular space, Bcl-2 family protein complex, nucleoplasm, extracellular region, cytosol, endoplasmic reticulum, nucleus, mitochondrion, cytoplasm and macromolecular complex (Supplementary Figure 1A).

Additionally, the molecular function mainly involved identical protein binding, ubiquitin protein ligase binding, protein binding, protein kinase binding, RNA polymerase II sequence-specific DNA binding transcription factor binding, BH3 domain binding, protein homodimerization activity, cytokine activity, enzyme binding and transmembrane receptor protein tyrosine kinase activity (Supplementary Figure 1B). There were 144 KEGG enrichment items in total, and the top 10 items were displayed according to p-values < 0.05 (Supplementary Table 9), included Hepatitis B, Prostate cancer, AGE-RAGE signaling pathway in diabetic complications, Hepatitis C, PI3K/AKT signaling pathway, HIF-1 signaling pathway, Kaposi sarcoma-associated herpesvirus infection (Figure 4D, 4E). Among them, the highest number of intersections genes were enriched in PI3K/AKT signaling pathway. From the enrichment study, *Atractylodes lancea* may exert its anti-cancer activity through PI3K/AKT signaling pathway.

### Molecular docking candidate gene screening

To obtain candidate genes for molecular docking, the target genes corresponding to each active ingredient of the drug were intersected with 73 genes, respectively, the component which intersected with the most genes was taken as the core ingredient of the drug. The results indicated that CID:5280445 (luteolin) had the greatest number of intersected genes (Table 1).

**Table 1:** The overlap of each active ingredient targets with 73 genes.

**PubChem_ID**	**Count**	**Gene**
CID:5280445	30	XDH,VRK1,GSTK1,ABCG2,BCL2,CASP9,MMP9,HMOX1,MAPK1,IL6,CASP3,VEGFA,NFKBIA,ERBB2,CDKN1A,CDK2,MCL1,BCL2L1,MET,ICAM1,IL10,INSR,TBK1,ADORA2A,PRKCE,EGFR,HMGB1,PTN,MAPK3,NFKB1
CID:5281703	21	CASP3,HIF1A,CTNNB1,MMP13,NFKB1,PRKCD,BCL2,MCL1,GSK3B,BBC3,CDKN1A,IL6,BAX,CCL26,MMP9,VEGFA,CFLAR,EGFR,SPP1,NEDD4L,MAPK1
CID:445639	14	MMP13,PPARD,PPARA,HMGCR,NTRK2,SERPINE1,CETP,MMP3,UCP2,ERBB2,SOAT1,AQP9,BCL2,STAT5B
CID:10228	12	SMAD1,CASP3,HMGCR,VEGFA,NFKB1,ABCB1,ERCC1,ICAM1,NFKBIA,CTNNB1,IL6,MAPK1
CID:440917	6	ADORA2A,BCL2,CASP3,CASP9,BAX,NFKB1
CID:5281416	6	CASP3,BCL2,CDK2,CDKN1A,RB1,MMP3
CID:985	6	CRTC2,IL10,GUCA2A,BCL2,YWHAZ,IL1A
CID:222284	5	CASP9,CASP3,BCL2,BAX,TGFB1
CID:31404	5	UCP1,HMOX1,BMP4,ICAM1,LBP
CID:5280450	5	PPARA,BCL2,ICAM1,FOS,NFKB1
CID:5321018	3	PGF,IL6,VEGFA
CID:6054	3	PPARA,BAX,STAT3
CID:931	3	FUS,BCL2,UCHL1
CID:31423	2	AHR,UGT1A6
CID:996	2	STAT3,AGTR2
CID:5281515	1	IL6
CID:5281522	1	PPARA
CID:5283349	1	RB1
CID:6989	1	BAX
CID:7410	1	TRPA1
CID:8468	1	HIF1A
CID:91457	1	TRPA1

### Survival, expression, and correlation analysis

**Figure 5:**
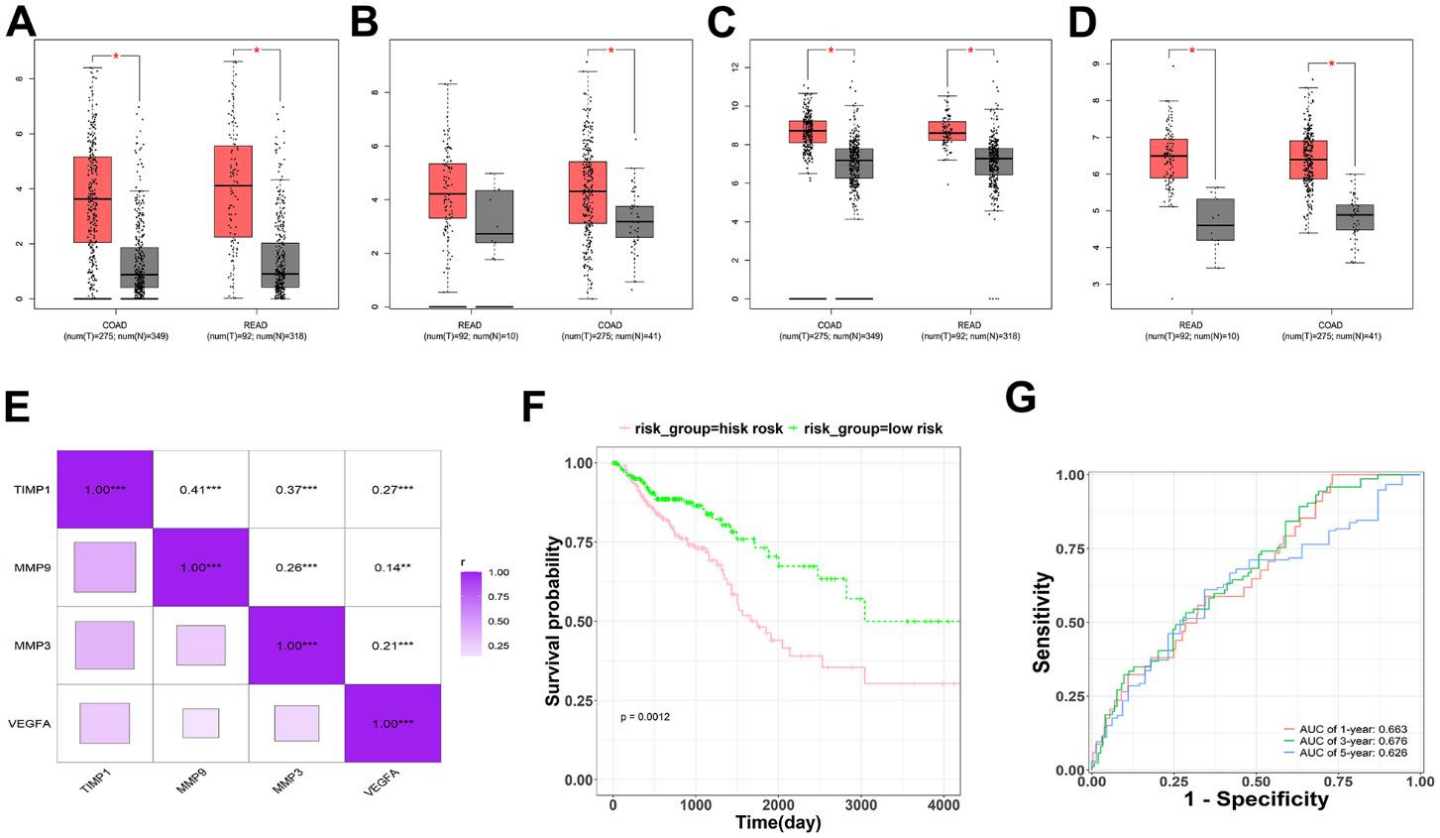
**Survival, expression, and correlation analysis of the hub-genes in CRC.** (**A**–**D**) Expression analysis for four genes (MMP3, MMP9, TIMP1 and VEGFA), red represents as Tumor, gray represents as Normal. (**E**) The correlation analysis for four genes (MMP3, MMP9, TIMP1 and VEGFA). (**F**) The KM survival plots for prognostic model which consist of four genes (MMP3, MMP9, TIMP1 and VEGFA). (**G**) The ROC curve analysis predicted this prognostic model at 1-, 3-, and 5-year survival rates.

To verify the expression of hub-genes in CRC, it was found that MMP3, MMP9, VEGFA, and TIMP1 were significantly differentially expressed in colorectal tumor, compared with normal tissue (Figure 5A–5D). And correlation analysis revealed that all four genes, MMP3, MMP9, VEGFA, and TIMP1, were significantly positively correlated, suggesting that these genes may have synergistic regulatory effects (Figure 5E). The prognostic risk model was constructed using MMP3, MMP9, VEGFA, and TIMP1, and the following formula was obtained: risk score = -0.09264*MMP3 - 0.06919*MMP9 + 0.44883*TIMP1 - 0.04962*VEGFA. In order to evaluate the clinical prognosis of the disease, Kaplan-Meier (KM) and ROC curve analysis was used, and the results showed that the prognostic model composed of MMP3, MMP9, VEGFA, and TIMP1 could effectively predict the prognostic risk of the patients (Figure 5F, 5G).

### Molecular docking analysis

**Figure 6:**
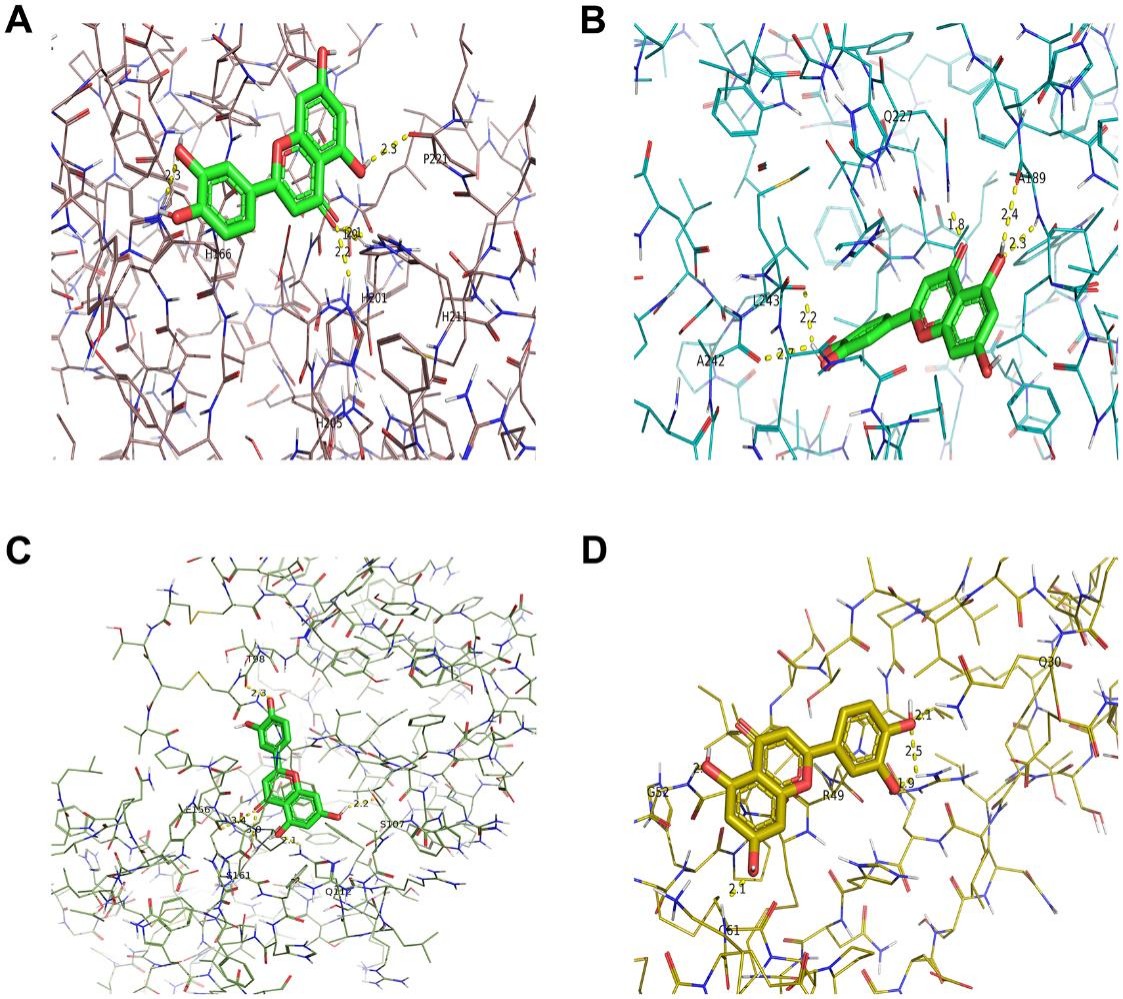
**Molecular models of luteolin binding to its predicted protein targets.** 3D receptor-ligand interaction diagram for the top four genes with the lowest binding energy for MMP3 (**A**), MMP9 (**B**), TIMP1 (**C**), VEGFA (**D**).

To explore whether luteolin, as the core active component of *Atractylodes lancea*, may interact with MMP3, MMP9, VEGFA, and TIMP1, the molecular docking of MMP3, MMP9, VEGFA, and TIMP1 with quercetin was carried out by using Autodock1.5.6 and visualization of docking results by PyMOL. Based on a binding energy of less than -5.0 kcal/mol and the formation of hydrogen bonds between receptor and ligands [32], the molecular docking results were shown in Table 2. The lower the binding energy, the more stable the structure. Among those target genes, MMP3, MMP9, VEGFA, and TIMP1 were found to have the lowest binding energy with luteolin. The 3D interaction between *Atractylodes lancea* and four target genes was shown in Figure 6.

**Table 2:** The results of molecular docking with luteolin.

**Target**	**PubChem_ID**	**Compound**	**Free binding energy (kcal/mol)**
MMP3	5280445	Luteolin	-7
MMP9			-10.5
TIMP1			-8.4
VEGFA			-5.9

### Luteolin represses the malignant phenotypes of CRC cells

**Figure 7:**
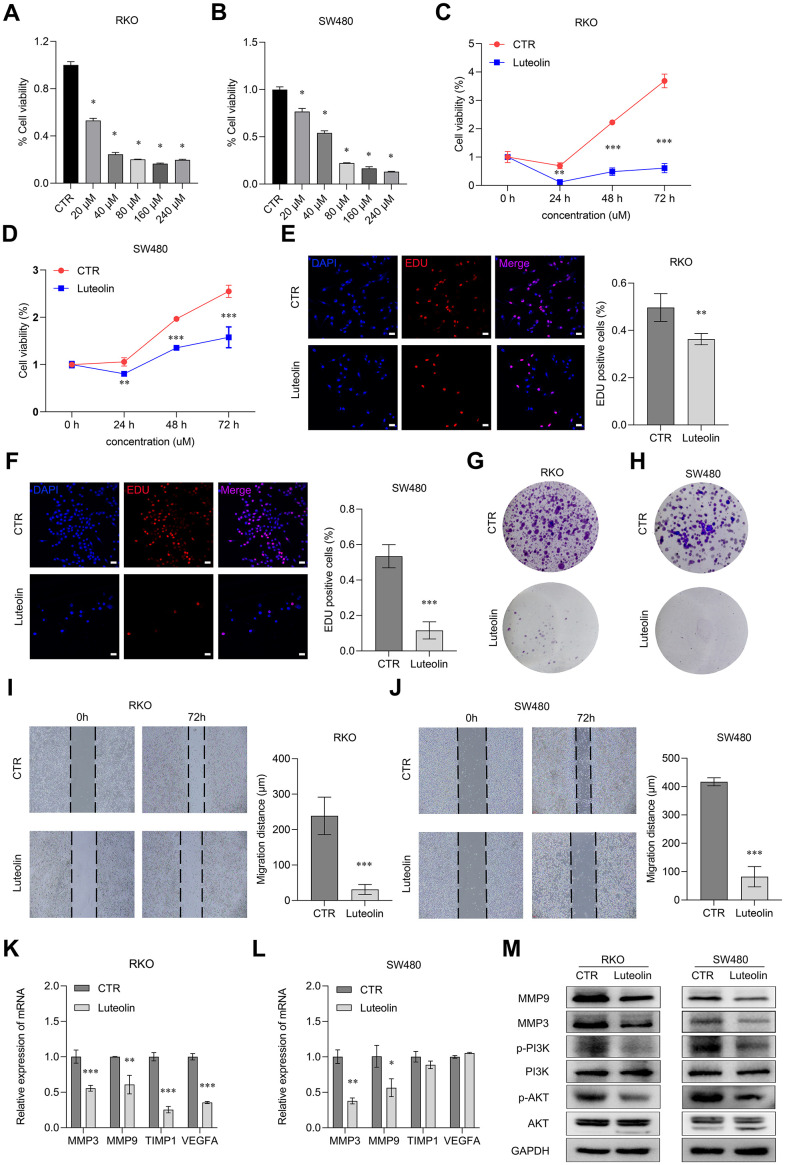
**Luteolin represses the malignant phenotypes of CRC cells.** (**A**, **B**) After treatment with different concentrations of luteolin (0, 20, 40, 80, 160, 200 μM) for 48 h, the cell viabilities of RKO and SW480 cells were determined by using CCK-8 assay. (**C**, **D**) After treatment with 40 μM luteolin or DMSO (control) for 24 h, 48 h and 72 h, the cell proliferation capacities of RKO and SW480 cells were determined using CCK-8. (**E**, **F**) After treatment with 40 μM luteolin or DMSO (control) for 24 h, the cell proliferation capacities of RKO and SW480 cells were determined using EdU assay. Bar = 20 μm. (**G**, **H**) After treatment with 40 μM luteolin or DMSO (control) for 2 weeks, the cell colonies were stained with crystal violet to display the effect of luteolin on proliferation of RKO and SW480 cells. (**I**, **J**) After treatment with 40 μM luteolin or DMSO (control) for 72 h, the migration distance of RKO and SW480 cells were assayed by wounding healing experiments. (**K**, **L**) After treatment with 40 μM luteolin or DMSO (control) for 24h, the expression level of MMP3, MMP9, TIMP1 and VEGFA in RKO and SW480 cells was detected by qRT-PCR assay. (**M**) After treatment with 40 μM luteolin or DMSO (control) for 24h, the expression levels of MMP9, MMP3, PI3K, p-PI3K, AKT and p-AKT in RKO and SW480 cells was analyzed by Western blot. Data are presented as mean ± SD, * *p* < 0.05, ** *p* < 0.01, *** *p* < 0.001 compared with the control group.

To further clarify the effects of luteolin on CRC cells, we performed a series of cellular phenotyping experiments. Before we started, we detected the expression levels of MMP3, MMP9, TIMP1, VEGFA in various colon cancer cell lines by qRT-PCR assay, and found that these genes were highly expressed in RKO and SW480, and we chose RKO and SW480 for the following experiments (Supplementary Figure 2). The cell viability was examined after treatment with different concentrations (0, 20, 40, 80, 160, 200 μM) of luteolin for 48 h by CCK-8 assay. As it was shown in Figure 7A–7D, luteolin inhibited the viability of CRC cells in a dose- and time-dependent manner. Consistent with the results of CCK-8 assays, the anti-proliferation effect of luteolin was confirmed by EdU assay and colony formation experiment (Figure 7E–7G). Wound healing experiments showed that luteolin inhibited cell motility (Figure 7I, 7J). According to the above informatics, qRT-PCR was used to detect the expression of MMP3, MMP9, TIMP1, VEGFA in CRC cells after luteolin treatment, and found that luteolin might down-regulate the expression of MMP3, MMP9, TIMP1, VEGFA in RKO cell lines, and only the expression of MMP3, MMP9 was decreased in SW480 (Figure 7K, 7L). We also found that the protein expression levels of MMP3, MMP9 and the activity of PI3K and AKT were inhibited after luteolin treatment (Figure 7M). In conclusion, luteolin could exert an anti-CRC effect through inhibiting PI3K/AKT signaling pathway.

## DISCUSSION

Despite advances in early detection and treatment, the incidence and mortality rates of CRC are still rising [33]. To develop novel prospective therapeutic targets and new drugs, the bioinformatics analysis was used to clarify the prognostic risk factors and targets of CRC. In our study, we predicted the target proteins associated with CRC progression that interacted with *Atractylodes lancea*, and elucidated the relevant mechanisms of anti-CRC effect by integrating information from public databases such as Genecards, GEO, TCGA, TCMSP and so on. Docking studies were performed to predict specific interactions between luteolin which was a core active ingredient of *Atractylodes lancea* and its predicted protein targets.

The overlapped genes shared by CRC and Atractylodes lance were obtained by pharmacology platform, disease database and expression datasets. Finally, 73 genes co-related with *Atractylodes lancea* and CRC and 10 hub genes were identified according to PPI network. The above hub genes were analyzed for GO and KEGG pathways enrichment. Notably, some of these biological processes analyses identified that *Atractylodes lancea* may act through oxidative stress in response to CRC. And KEGG analysis of *Atractylodes lancea*’s anti-CRC activity revealed several related signaling pathways, including PI3K/AKT signaling pathway, HIF-1 signaling pathway. These signaling pathways are interconnected and known to be associated with oxidative stress [34–36]. Thus, multiple signaling pathways may be involved and contributed to *Atractylodes lancea*’s overall anti-CRC activity.

The molecular docking results of luteolin which as the core ingredients of *Atractylodes lancea* showed that hydrogen bonding was the main forms of interaction. And four genes (MMP3, MMP9, TIMP1 and VEGFA) which have the lowest binding energy to combine with luteolin were found to be involved in CRC progression. Expression analysis showed that MMP3, MMP9, TIMP1 and VEGFA were upregulated in tumor samples. Correlation, survival and ROC curve analysis between those genes supported these results, inferring that MMP3, MMP9 and VEGFA were negatively associated with prognostic risk, and TIMP1 was positively associated with prognostic risk. We inferred that luteolin affects the malignant phenotype of CRC by regulating the expression of MMP3, MMP9, TIMP1 and VEGFA.

MMPs, which participated to degrade extracellular matrix, plays an important role in CRC metastasis [37]. Degradation of extracellular matrix was essential for tumor invasion and metastasis [38]. Based on using immortalized epithelial cell lines or transgenic mouse models, MMP3 plays an important role in tumor initiation [39]. As well as MMP3, increasing MMP9 transcription enhanced cell invasion [40]. MMP3 and MMP9 expression have been proposed to be a biological predictor in CRC [41]. Previous studies have reported that *Atractylodes lancea* inhibited osteoarthritis by suppressing the expression and activity of MMP3 [42], and *Atractylodes lancea* inhibited migration and invasion of hepatocellular carcinoma cells by decreasing the expression level of MMP9 [43]. Consistent with those studies, we also found that *Atractylodes lancea* suppresses the proliferation of CRC cells, accompanied with a decrease in MMP3 and MMP9 expression.

TIMP1 was expressed at a high level in CRC, and decreased expression level of TIMP1 was significantly associated with poor prognosis of CRC [44]. Down-regulation of TIMP1 inhibited the proliferation and metastasis of colon cancer, but promoted apoptosis through the focal adhesion PI3K/AKT and mitogen-activated protein kinase pathway [45]. High level of TIMP1 was associated with a short overall survival time for CRC patients, especially the plasma level of TIMP1, therefore the expression of TIMP1 could be a potential prognostic indicator [46]. In our research, the expression of TIMP1 in RKO cells was inhibited, but the expression of TIMP1 in SW480 cells has no changes after *Atractylodes lancea* treatment. The reason for this phenomenon may be due to the fact that different tumor cells have different genetic backgrounds.

VEGFA, an endothelial cell-specific mitogen, which participate in physiological and pathological angiogenesis to promote endothelial cell growth, migration, differentiation and vascular permeability [47]. Previous studies have shown that the expression level of VEGF was increased in CRC tissue samples and was associated with poor clinical prognosis [48]. Inhibited VEGFA expression significantly suppressed tumor angiogenesis in CRC cells [49]. VEGFA plays a crucial role in angiogenesis in CRC and has become a major target for anti-angiogenic drugs [50]. It has been reported that codonolactone, a component of *Atractylodes lancea*, was able to inhibit angiogenesis in blast cancer by decreasing the expression level of VEGF [51]. And our results showed that under luteolin treatment, the expression of VEGFA in RKO was decreased. And it could be a candidate for anti-angiogenesis drugs in CRC. The reason for this may be the result in the anti-cancer effects of *Atractylodes lancea* in different cancers with different components.

Many biological activities, including cell proliferation, differentiation, migration, and death, were regulated by PI3K/AKT signaling pathways. Notably, genome-wide association studies have confirmed the PI3K/AKT signaling pathway as one of the genetic markers most closely associated with CRC. In CRC, PI3K/AKT was associated with tumor proliferation, migration, tumor progression and poor prognosis [52]. Our research showed that PI3K/AKT signaling was inhibited under luteolin treatment. A series of studies have been reported that MMP3 [53], MMP9 [54], TIMP1 [45] and VEGFA [55] could be regulated by PI3K/AKT signaling pathway. Therefore, *Atractylodes lancea* inhibited the proliferation and migration of CRC cells by suppressing the expression of MMP3, MMP9, TIMP1 and VEGFA probably through PI3K/AKT signaling pathway.

## CONCLUSIONS

Our study analyzed the role, core active component and mechanisms of *Atractylodes lancea* in CRC using network pharmacology and phenotyping experiments. Our findings revealed that the anti-CRC effect of *Atractylodes lancea* via an array of targets and pathways. *Atractylodes lancea* may have a therapeutic role in CRC through inhibiting cell proliferation and migration by targeting the hub genes (MMP3, MMP9, TIMP1 and VEGFA) though PI3K/AKT signaling pathway. Despite clarifying the mechanisms of *Atractylodes lancea* with hub genes which need more pre- and clinical experiments, our findings still offer a reference for further investigation of the anti-tumor effect of *Atractylodes lancea* in CRC.

## Supplementary Material

Supplementary Figures

Supplementary Tables 1 and 2

Supplementary Table 3

Supplementary Table 4

Supplementary Table 5

Supplementary Table 6

Supplementary Table 7

Supplementary Table 8

Supplementary Table 9
