# Supplementary material for: Elucidation of the anti-colorectal cancer mechanism of Atractylodes lancea by network pharmacology and experimental verification
Source: Aging (Albany NY). 2024 Aug 22;16(16):12008–28. doi: 10.18632/aging.206075 (PMC11386916; doi:10.18632/aging.206075)
Supplement: Supplementary Figures [file aging-16-206075-s001.pdf]

SUPPLEMENTARY MATERIALS

Supplementary Figures

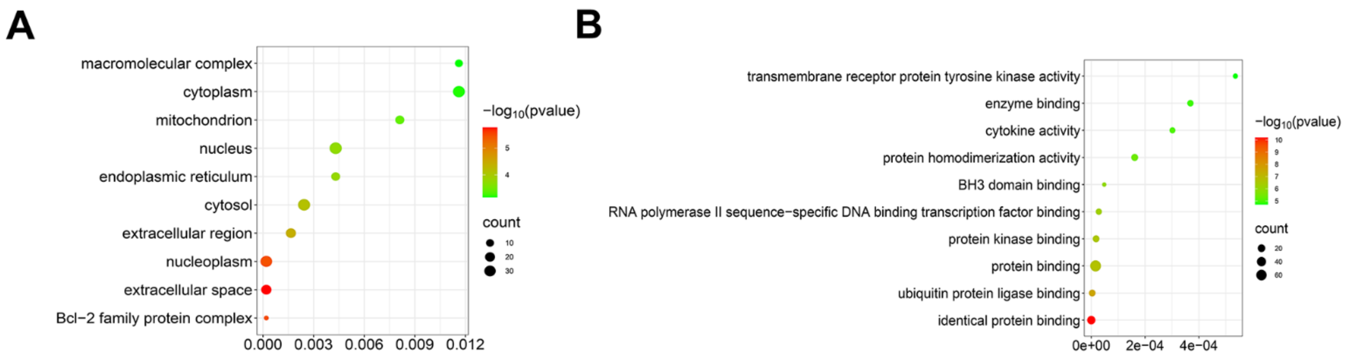

Supplementary Figure 1. GO enrichment analysis shows the cellular components and molecular function of 73 overlapping genes. (**A**) GO enrichment analysis shows the intersection target genes in cellular components localization and the top 10 results were displayed. (**B**) GO enrichment analysis shows the intersection target genes in molecular function localization and the top 10 results were displayed.

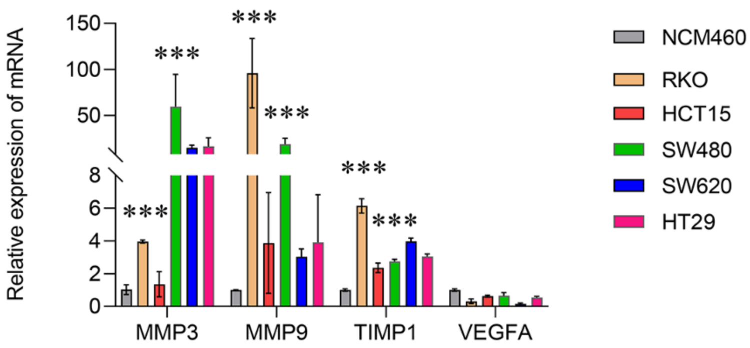

Supplementary Figure 2. The expression levels of MMP3, MMP9, TIMP1 and VEGFA in CRC cell lines were detected by qRT-PCR.
