# Supplementary material for: Elucidation of the anti-colorectal cancer mechanism of Atractylodes lancea by network pharmacology and experimental verification
Source: Aging (Albany NY). 2024 Aug 22;16(16):12008–28. doi: 10.18632/aging.206075 (PMC11386916; doi:10.18632/aging.206075)
Supplement: Supplementary Tables 1 and 2 [file aging-16-206075-s002.pdf]

## SUPPLEMENTARY TABLES

**Supplementary Table 1. Sequence information for primers used in qRT-PCR.**

| Gene | Forward sequence | Reverse sequence |
|---|---|---|
| *18S* | 5'-AACCCGTTGAACCCCATT -3' | 5'-CCATCCAATCGGTAGTAGCG -3' |
| *MMP3* | 5'- CGGTTCCGCCTGTCTCAAG-3' | 5'- CGCCAAAAGTGCCTGTCTT-3' |
| *MMP9* | 5'- GGGACGCAGACATCGTCATC-3' | 5'- TCGTCATCGTCGAAATGGGC-3' |
| *TIMP1* | 5'- AGAGTGTCTGCGGATACTTCC-3' | 5'- CCAACAGTGTAGGTCTTGGTG-3' |
| *VEGFA* | 5'- AGGGCAGAATCATCACGAAGT-3' | 5'- AGGGTCTCGATTGGATGGCA-3' |

**Supplementary Table 2. The active ingredients of *Atractylodes lancea*.**

| PubChem_ID | Cpd_name |
|---|---|
| CID:10228 | Osthole |
| CID:6989 | Thymol |
| CID:8468 | Vanillic Acid |
| CID:5281703 | Wogonin |
| CID:931 | Naphthalene |
| CID:996 | Phenol |
| CID:985 | Palmitic Acid |
| CID:1140 | Toluene |
| CID:7095 | Biphenyl |
| CID:7410 | Acetophenone |
| CID:31423 | Pyrene |
| CID:31404 | Butylated Hydroxytoluene |
| CID:445639 | Oleic Acid |
| CID:222284 | Beta-Sitosterol |
| CID:853433 | Isoeugenol |
| CID:5280445 | Luteolin |
| CID:5280450 | Linoleic Acid |
| CID:5281416 | Esculetin |
| CID:6054 | Phenylethyl Alcohol |
| CID:440917 | D-Limonene |
| CID:227829 | Guaiol |
| CID:5281522 | Isocaryophyllene |
| CID:91457 | beta-Eudesmol |
| CID:5283349 | 2,4-Decadienal |
| CID:5281520 | Alpha-Humulone |
| CID:5281515 | Caryophyllene |
| CID:7044 | Decahydronaphthalene |
| CID:7461 | gamma-Terpinene |
| CID:31272 | Butyl acetate |
| CID:60961 | Adenosine |
| CID:10878761 | Hinesol |
| CID:675 | 5,6-Dimethylbenzimidazole |
| CID:676946 | p-Coumaric acid ethyl ester |
| CID:7362 | Furfural |

| | |
|---|---|
| CID:460 | Guaiacol |
| CID:6885 | Phthalide |
| CID:5280794 | Stigmasterol |
| CID:5541 | Triacetin |
| CID:5281426 | Umbelliferone |
| CID:5283345 | 2-Decenal |
| CID:442353 | beta-Chamigrene |
| CID:5283316 | 2-Heptenal |
| CID:5283356 | 2-Undecenal |
| CID:3080635 | Atractylon |
| CID:5321047 | Atractylodin |
| CID:10012964 | Atractylodinol |
| CID:10929902 | Atractyloside I |
| CID:71448957 | Atractyloside G |
| CID:71307451 | Atractyloside A |
| CID:12366272 | Cirsiumaldehyde |
| CID:14079045 | Icariside F2 |
| CID:71448952 | Atractyloside B |
| CID:71448953 | Atractyloside C |
| CID:71448954 | Atractyloside D |
| CID:71448955 | Atractyloside E |
| CID:12138536 | α-Hexylcinnamaldehyde |
| CID:10353528 | Agarospirol |
| CID:1549992 | Bisabolol |
| CID:71448961 | 3beta-Hydroxyatractylon |
| CID:78249 | Limonene |
| CID:5317844 | alpha-Guaiene |
| CID:3084961 | Wogonoside |
| CID:5321018 | Atractylenolide I |
| CID:14420566 | Parthenolide |
| CID:70697841 | Gaillardin |
| CID:12309818 | beta-Eudesmol cis epimer |
| CID:5356634 | Stigmastenone |
| CID:6429301 | (Z)-caryophyllene |
| CID:92138 | Elemol |
| CID:14448070 | Atractylenolide II |
| CID:13986099 | 3,5-Bis(tert-butyl) benzaldehyde |
| CID:6451614 | Stigmastanol |
