# Supplementary material for: Elucidation of the anti-colorectal cancer mechanism of Atractylodes lancea by network pharmacology and experimental verification
Source: Aging (Albany NY). 2024 Aug 22;16(16):12008–28. doi: 10.18632/aging.206075 (PMC11386916; doi:10.18632/aging.206075)
Supplement: Supplementary Table 3 [file aging-16-206075-s003.docx]

**Supplementary Table 3. Predicted targets of the potential bioactive compounds of *Atractylodes lancea*.**

| Gene name | Uniprot_ID | Cpd_name | Pubchem_ID |
| --- | --- | --- | --- |
| PTK2  FASN  BMP2  SMAD1  CASP3  HMGCR  VEGFA  NFKB1  ABCB1  ERCC1  DIABLO  DGAT1  ICAM1  NFKBIA  CTNNB1  VCAM1  PTGS2  AKT1  APP  MPO  TNF  IL6  BACE1  MAPK1  MAP2  CYP2E1  GFAP  SRD5A2  BDNF  CREB1  CCL3  CAMK4  CASP9  CASP3  PRKAA2  CASP8  BCL2  BAX  TGFB1  MAP2  PRKCA  EDN1  RAD51  PLSCR1  PSME1  ADIPOR2  UCP1  EIF2AK2  SFTPB  PECAM1  HMOX1  KDR  BMP4  ICAM1  LBP  CXCL8  AHR  CXADR  UGT1A1  UGT1A6  NPY  ADORA2A  BCL2  TP53  CASP3  CASP9  BAX  IL1B  MPO  NFKB1  MPO  MMP13  NOS2  PPARD  PLG  PPARA  PPARG  MSI1  PRSS1  FABP5  HMGCR  BDNF  NTRK2  CRP  PDX1  SLC2A2  INS  PON1  PAM  GAP43  EDN1  SERPINE1  GCG  CETP  DNPEP  MMP3  LALBA  UCP2  UCP3  CCK  FABP1  RBP2  ERBB2  SOAT1  AQP3  AQP9  BCL2  GLP1R  STAT5A  STAT5B  TOP2A  XDH  CDK9  FASN  NFE2L2  VRK1  HSP90AA1  GSTK1  ERG  ABCC2  ABCG2  MAPK14  HDAC9  MTOR  FLT3  ANO1  BCL2  MAOA  IL17A  GAK  MMP2  CASP9  TOP1  MMP9  XIAP  TP53  TYR  HMOX1  MAPK1  IL6  JUN  CASP3  VEGFA  AKT1  NFKBIA  PTGS2  PTGES  ERBB2  CDKN1A  IL4  CDK4  CDK2  CCND1  CCNB1  MCL1  BCL2L1  MDM2  MET  TNF  NOS2  ICAM1  IL10  IFNG  INSR  SLC2A4  MMP1  TBK1  MAPK8  AURKB  ADORA2A  PRKCE  ACHE  ADORA1  SRC  EGFR  ZHX2  PARP1  CLDN2  SMAD3  HMGB1  ESR1  TERT  SIRT3  PTN  MAPK3  NFKB1  PPARA  PLA2G7  POMC  LCN2  CCL2  BCL2  ICAM1  NPPA  SCD  FOS  NPPB  NFKB1  JUN  NFATC4  CASP3  BCL2  CDK2  CDK4  CDKN1A  KEAP1  MMP1  RB1  MMP3  ACE2  CNR2  IL6  TNF  IL1B  CNR2  PPARA  CYP1A2  CASP3  HIF1A  CTNNB1  MMP13  NFKB1  MUC5AC  CYP2C19  RELA  PRKCD  TP53  MMP1  BCL2  PTGS2  MCL1  CCL2  CDK9  NOS2  GSK3B  CCND1  BBC3  CDKN1A  IL6  CXCL8  BAX  KDR  AKT1  PTGS1  FN1  JUN  F3  CCL26  MMP9  CDKN1B  MAPK11  TNF  ERN1  VEGFA  CFLAR  EGFR  SPP1  NEDD4L  CDK4  TRAF2  MAPK1  TNF  F3  CD36  CDK4  RB1  CCND1  CDKN1B  NOS2  ALOX5  PGF  TLR4  TNF  IL6  VEGFA  IL1B  PPARA  BAX  STAT3  ELANE  BAX  CXCR4  TRPA1  TYR  TYRP1  HIF1A  NOS3  CA3  IL2  RELA  TRPA1  CYP1A1  MPO  FUS  BCL2  CXCL8  UCHL1  TLR2  CRTC2  IL10  TNF  APOM  GUCA2A  PTEN  BCL2  ABHD15  YWHAZ  IDE  IL1A  ACE  STAT3  MB  AGTR2 | Q05397  P49327  P12643  Q15797  P42574  P04035  P15692  P19838  P08183  P07992  Q9NR28  O75907  P05362  P25963  P35222  P19320  P35354  P31749  P05067  P05164  P01375  P05231  P56817  P28482  P11137  P05181  P14136  P31213  P23560  P16220  P10147  Q16566  P55211  P42574  P54646  Q14790  P10415  Q07812  P01137  P11137  P17252  P05305  Q06609  O15162  Q06323  Q86V24  P25874  P19525  P07988  P16284  P09601  P35968  P12644  P05362  P18428  P10145  P35869  P78310  P22309  P19224  P01303  P29274  P10415  P04637  P42574  P55211  Q07812  P01584  P05164  P19838  P05164  P45452  P35228  Q03181  P00747  Q07869  P37231  O43347  P07477  Q01469  P04035  P23560  Q16620  P02741  P52945  P11168  P01308  P27169  P19021  P17677  P05305  P05121  P01275  P11597  Q9ULA0  P08254  P00709  P55851  P55916  P06307  P07148  P50120  P04626  P35610  Q92482  O43315  P10415  P43220  P42229  P51692  P11388  P47989  P50750  P49327  Q16236  Q99986  P07900  Q9Y2Q3  P11308  Q92887  Q9UNQ0  Q16539  Q9UKV0  P42345  P36888  Q5XXA6  P10415  P21397  Q16552  O14976  P08253  P55211  P11387  P14780  P98170  P04637  P14679  P09601  P28482  P05231  P05412  P42574  P15692  P31749  P25963  P35354  O14684  P04626  P38936  P05112  P11802  P24941  P24385  P14635  Q07820  Q07817  Q00987  P08581  P01375  P35228  P05362  P22301  P01579  P06213  P14672  P03956  Q9UHD2  P45983  Q96GD4  P29274  Q02156  P22303  P30542  P12931  P00533  Q9Y6X8  P09874  P57739  P84022  P09429  P03372  O14746  Q9NTG7  P21246  P27361  P19838  Q07869  Q13093  P01189  P80188  P13500  P10415  P05362  P01160  O00767  P01100  P16860  P19838  P05412  Q14934  P42574  P10415  P24941  P11802  P38936  Q14145  P03956  P06400  P08254  Q9BYF1  P34972  P05231  P01375  P01584  P34972  Q07869  P05177  P42574  Q16665  P35222  P45452  P19838  P98088  P33261  Q04206  Q05655  P04637  P03956  P10415  P35354  Q07820  P13500  P50750  P35228  P49841  P24385  Q9BXH1  P38936  P05231  P10145  Q07812  P35968  P31749  P23219  P02751  P05412  P13726  Q9Y258  P14780  P46527  Q15759  P01375  O75460  P15692  O15519  P00533  P10451  Q96PU5  P11802  Q12933  P28482  P01375  P13726  P16671  P11802  P06400  P24385  P46527  P35228  P09917  P49763  O00206  P01375  P05231  P15692  P01584  Q07869  Q07812  P40763  P08246  Q07812  P61073  O75762  P14679  P17643  Q16665  P29474  P07451  P60568  Q04206  O75762  P04798  P05164  P35637  P10415  P10145  P09936  O60603  Q53ET0  P22301  P01375  O95445  Q02747  P60484  P10415  Q6UXT9  P63104  P14735  P01583  P12821  P40763  P02144  P50052 | Osthole  Osthole  Osthole  Osthole  Osthole  Osthole  Osthole  Osthole  Osthole  Osthole  Osthole  Osthole  Osthole  Osthole  Osthole  Osthole  Osthole  Osthole  Osthole  Osthole  Osthole  Osthole  Osthole  Osthole  Toluene  Toluene  Toluene  Toluene  Toluene  Toluene  Toluene  Toluene  Beta-Sitosterol  Beta-Sitosterol  Beta-Sitosterol  Beta-Sitosterol  Beta-Sitosterol  Beta-Sitosterol  Beta-Sitosterol  Beta-Sitosterol  Beta-Sitosterol  Beta-Sitosterol  Guaiol  Wogonoside  Butylated Hydroxytoluene  Butylated Hydroxytoluene  Butylated Hydroxytoluene  Butylated Hydroxytoluene  Butylated Hydroxytoluene  Butylated Hydroxytoluene  Butylated Hydroxytoluene  Butylated Hydroxytoluene  Butylated Hydroxytoluene  Butylated Hydroxytoluene  Butylated Hydroxytoluene  Pyrene  Pyrene  Pyrene  Pyrene  Pyrene  Limonene  Limonene  Limonene  Limonene  Limonene  Limonene  Limonene  Limonene  Limonene  Limonene  Oleic Acid  Oleic Acid  Oleic Acid  Oleic Acid  Oleic Acid  Oleic Acid  Oleic Acid  Oleic Acid  Oleic Acid  Oleic Acid  Oleic Acid  Oleic Acid  Oleic Acid  Oleic Acid  Oleic Acid  Oleic Acid  Oleic Acid  Oleic Acid  Oleic Acid  Oleic Acid  Oleic Acid  Oleic Acid  Oleic Acid  Oleic Acid  Oleic Acid  Oleic Acid  Oleic Acid  Oleic Acid  Oleic Acid  Oleic Acid  Oleic Acid  Oleic Acid  Oleic Acid  Oleic Acid  Oleic Acid  Oleic Acid  Oleic Acid  Oleic Acid  Oleic Acid  Oleic Acid  Luteolin  Luteolin  Luteolin  Luteolin  Luteolin  Luteolin  Luteolin  Luteolin  Luteolin  Luteolin  Luteolin  Luteolin  Luteolin  Luteolin  Luteolin  Luteolin  Luteolin  Luteolin  Luteolin  Luteolin  Luteolin  Luteolin  Luteolin  Luteolin  Luteolin  Luteolin  Luteolin  Luteolin  Luteolin  Luteolin  Luteolin  Luteolin  Luteolin  Luteolin  Luteolin  Luteolin  Luteolin  Luteolin  Luteolin  Luteolin  Luteolin  Luteolin  Luteolin  Luteolin  Luteolin  Luteolin  Luteolin  Luteolin  Luteolin  Luteolin  Luteolin  Luteolin  Luteolin  Luteolin  Luteolin  Luteolin  Luteolin  Luteolin  Luteolin  Luteolin  Luteolin  Luteolin  Luteolin  Luteolin  Luteolin  Luteolin  Luteolin  Luteolin  Luteolin  Luteolin  Luteolin  Luteolin  Luteolin  Luteolin  Luteolin  Luteolin  Linoleic Acid  Linoleic Acid  Linoleic Acid  Linoleic Acid  Linoleic Acid  Linoleic Acid  Linoleic Acid  Linoleic Acid  Linoleic Acid  Linoleic Acid  Linoleic Acid  Linoleic Acid  Linoleic Acid  Linoleic Acid  Esculetin  Esculetin  Esculetin  Esculetin  Esculetin  Esculetin  Esculetin  Esculetin  Esculetin  Esculetin  Caryophyllene  Caryophyllene  Alpha-Humulone  Alpha-Humulone  Isocaryophyllene  Isocaryophyllene  Wogonin  Wogonin  Wogonin  Wogonin  Wogonin  Wogonin  Wogonin  Wogonin  Wogonin  Wogonin  Wogonin  Wogonin  Wogonin  Wogonin  Wogonin  Wogonin  Wogonin  Wogonin  Wogonin  Wogonin  Wogonin  Wogonin  Wogonin  Wogonin  Wogonin  Wogonin  Wogonin  Wogonin  Wogonin  Wogonin  Wogonin  Wogonin  Wogonin  Wogonin  Wogonin  Wogonin  Wogonin  Wogonin  Wogonin  Wogonin  Wogonin  Wogonin  Wogonin  Wogonin  Wogonin  2,4-Decadienal  2,4-Decadienal  2,4-Decadienal  2,4-Decadienal  2,4-Decadienal  2,4-Decadienal  2,4-Decadienal  Atractylenolide I  Atractylenolide I  Atractylenolide I  Atractylenolide I  Atractylenolide I  Atractylenolide I  Atractylenolide I  Atractylenolide I  Phenylethyl Alcohol  Phenylethyl Alcohol  Phenylethyl Alcohol  Thymol  Thymol  Biphenyl  Acetophenone  Vanillic Acid  Vanillic Acid  Vanillic Acid  Vanillic Acid  Vanillic Acid  Isoeugenol  Isoeugenol  Beta-Eudesmol  Naphthalene  Naphthalene  Naphthalene  Naphthalene  Naphthalene  Naphthalene  Palmitic Acid  Palmitic Acid  Palmitic Acid  Palmitic Acid  Palmitic Acid  Palmitic Acid  Palmitic Acid  Palmitic Acid  Palmitic Acid  Palmitic Acid  Palmitic Acid  Palmitic Acid  Phenol  Phenol  Phenol  Phenol | CID:10228  CID:10228  CID:10228  CID:10228  CID:10228  CID:10228  CID:10228  CID:10228  CID:10228  CID:10228  CID:10228  CID:10228  CID:10228  CID:10228  CID:10228  CID:10228  CID:10228  CID:10228  CID:10228  CID:10228  CID:10228  CID:10228  CID:10228  CID:10228  CID:1140  CID:1140  CID:1140  CID:1140  CID:1140  CID:1140  CID:1140  CID:1140  CID:222284  CID:222284  CID:222284  CID:222284  CID:222284  CID:222284  CID:222284  CID:222284  CID:222284  CID:222284  CID:227829  CID:3084961  CID:31404  CID:31404  CID:31404  CID:31404  CID:31404  CID:31404  CID:31404  CID:31404  CID:31404  CID:31404  CID:31404  CID:31423  CID:31423  CID:31423  CID:31423  CID:31423  CID:440917  CID:440917  CID:440917  CID:440917  CID:440917  CID:440917  CID:440917  CID:440917  CID:440917  CID:440917  CID:445639  CID:445639  CID:445639  CID:445639  CID:445639  CID:445639  CID:445639  CID:445639  CID:445639  CID:445639  CID:445639  CID:445639  CID:445639  CID:445639  CID:445639  CID:445639  CID:445639  CID:445639  CID:445639  CID:445639  CID:445639  CID:445639  CID:445639  CID:445639  CID:445639  CID:445639  CID:445639  CID:445639  CID:445639  CID:445639  CID:445639  CID:445639  CID:445639  CID:445639  CID:445639  CID:445639  CID:445639  CID:445639  CID:445639  CID:445639  CID:5280445  CID:5280445  CID:5280445  CID:5280445  CID:5280445  CID:5280445  CID:5280445  CID:5280445  CID:5280445  CID:5280445  CID:5280445  CID:5280445  CID:5280445  CID:5280445  CID:5280445  CID:5280445  CID:5280445  CID:5280445  CID:5280445  CID:5280445  CID:5280445  CID:5280445  CID:5280445  CID:5280445  CID:5280445  CID:5280445  CID:5280445  CID:5280445  CID:5280445  CID:5280445  CID:5280445  CID:5280445  CID:5280445  CID:5280445  CID:5280445  CID:5280445  CID:5280445  CID:5280445  CID:5280445  CID:5280445  CID:5280445  CID:5280445  CID:5280445  CID:5280445  CID:5280445  CID:5280445  CID:5280445  CID:5280445  CID:5280445  CID:5280445  CID:5280445  CID:5280445  CID:5280445  CID:5280445  CID:5280445  CID:5280445  CID:5280445  CID:5280445  CID:5280445  CID:5280445  CID:5280445  CID:5280445  CID:5280445  CID:5280445  CID:5280445  CID:5280445  CID:5280445  CID:5280445  CID:5280445  CID:5280445  CID:5280445  CID:5280445  CID:5280445  CID:5280445  CID:5280445  CID:5280445  CID:5280450  CID:5280450  CID:5280450  CID:5280450  CID:5280450  CID:5280450  CID:5280450  CID:5280450  CID:5280450  CID:5280450  CID:5280450  CID:5280450  CID:5280450  CID:5280450  CID:5281416  CID:5281416  CID:5281416  CID:5281416  CID:5281416  CID:5281416  CID:5281416  CID:5281416  CID:5281416  CID:5281416  CID:5281515  CID:5281515  CID:5281520  CID:5281520  CID:5281522  CID:5281522  CID:5281703  CID:5281703  CID:5281703  CID:5281703  CID:5281703  CID:5281703  CID:5281703  CID:5281703  CID:5281703  CID:5281703  CID:5281703  CID:5281703  CID:5281703  CID:5281703  CID:5281703  CID:5281703  CID:5281703  CID:5281703  CID:5281703  CID:5281703  CID:5281703  CID:5281703  CID:5281703  CID:5281703  CID:5281703  CID:5281703  CID:5281703  CID:5281703  CID:5281703  CID:5281703  CID:5281703  CID:5281703  CID:5281703  CID:5281703  CID:5281703  CID:5281703  CID:5281703  CID:5281703  CID:5281703  CID:5281703  CID:5281703  CID:5281703  CID:5281703  CID:5281703  CID:5281703  CID:5283349  CID:5283349  CID:5283349  CID:5283349  CID:5283349  CID:5283349  CID:5283349  CID:5321018  CID:5321018  CID:5321018  CID:5321018  CID:5321018  CID:5321018  CID:5321018  CID:5321018  CID:6054  CID:6054  CID:6054  CID:6989  CID:6989  CID:7095  CID:7410  CID:8468  CID:8468  CID:8468  CID:8468  CID:8468  CID:853433  CID:853433  CID:91457  CID:931  CID:931  CID:931  CID:931  CID:931  CID:931  CID:985  CID:985  CID:985  CID:985  CID:985  CID:985  CID:985  CID:985  CID:985  CID:985  CID:985  CID:985  CID:996  CID:996  CID:996  CID:996 |
