# Supplementary material for: Elucidation of the anti-colorectal cancer mechanism of Atractylodes lancea by network pharmacology and experimental verification
Source: Aging (Albany NY). 2024 Aug 22;16(16):12008–28. doi: 10.18632/aging.206075 (PMC11386916; doi:10.18632/aging.206075)
Supplement: Supplementary Table 7 [file aging-16-206075-s007.pdf]

Supplementary Table 7. Predicted targets of the potential bioactive compounds of *Atractylodes lancea* (73 genes).

geneName	source	uniprot
CTNNB1	DigSee	P35222
BCL2L1	DigSee	Q07817
STAT3	DigSee	P40763
EGFR	DigSee	P00533
BCL2	DigSee	P10415
HIF1A	DigSee	Q16665
MMP9	DigSee	P14780
VEGFA	DigSee	P15692
NFKB1	DigSee	P19838
SERPINE1	DigSee	P05121
IL10	DigSee	P22301
ABCB1	DigSee	P08183
CDKN1A	DigSee	P38936
MCL1	DigSee	Q07820
GSK3B	DigSee	P49841
BAX	DigSee	Q07812
UCHL1	DigSee	P09936
MET	DigSee	P08581
ERBB2	DigSee	P04626
CDK2	DigSee	P24941
XDH	DigSee	P47989
PPARA	DigSee	Q07869
PPARD	DigSee	Q03181
IL6	DigSee	P05231
SPP1	DigSee	P10451
TGFB1	DigSee	P01137
ERCC1	DigSee	P07992
NFKBIA	DigSee	P25963
PGF	DigSee	P49763
HMOX1	DigSee	P09601
HMGB1	DigSee	P09429
BMP4	DigSee	P12644
ICAM1	DigSee	P05362
MAPK3	DigSee	P27361
FOS	DigSee	P01100
MAPK1	DigSee	P28482
INSR	DigSee	P06213
FUS	DigSee	P35637
CASP3	DigSee	P42574
ABCG2	DigSee	Q9UNQ0
MMP3	DigSee	P08254
GUCA2A	DigSee	Q02747
GSTK1	DigSee	Q9Y2Q3
NTRK2	DigSee	Q16620
RB1	DigSee	P06400
CFLAR	DigSee	O15519

CASP9	DigSee	P55211
HMGCR	DigSee	P04035
IL1A	DigSee	P01583
AHR	DigSee	P35869
SOAT1	DigSee	P35610
UCP2	DigSee	P55851
PTN	DigSee	P21246
BBC3	OMIM	Q9BXH1
UGT1A6	GeneCards	P19224
MMP13	GeneCards	P45452
TBK1	GeneCards	Q9UHD2
LBP	GeneCards	P18428
AGTR2	GeneCards	P50052
PRKCE	GeneCards	Q02156
UCP1	GeneCards	P25874
CCL26	GeneCards	Q9Y258
PRKCD	GeneCards	Q05655
NEDD4L	GeneCards	Q96PU5
SMAD1	GeneCards	Q15797
YWHAZ	GeneCards	P63104
STAT5B	GeneCards	P51692
AQP9	GeneCards	O43315
CETP	GeneCards	P11597
CRTC2	GeneCards	Q53ET0
ADORA2A	GeneCards	P29274
TRPA1	GeneCards	O75762
VRK1	GeneCards	Q99986
